# A Rare Case of Gingival Osseous Choristoma Misdiagnosed as Fibroma

**DOI:** 10.1155/crid/9494235

**Published:** 2026-04-29

**Authors:** Yilin Wang, Chu Xu, Ting Chen

**Affiliations:** ^1^ Department of Stomatology, Nanfang Hospital, Southern Medical University, Guangzhou, Guangdong, China, fimmu.com

**Keywords:** bone, case report, choristoma, gingiva, heterotopic, osseous

## Abstract

Osseous choristoma is a rare, nonneoplastic lesion defined by the presence of organized lamellar bone within soft tissue. It most frequently affects the dorsal tongue. Gingival involvement is extremely rare, with only nine cases documented in the English‐language literature. This report describes a 20‐year‐old woman who presented with a well‐circumscribed, painless, firm, and sessile nodule on the buccal gingiva of the left posterior mandible. Panoramic radiography revealed no pathological alterations. The initial clinical impression was fibroma. Surgical excision was performed. Histopathology revealed well‐demarcated nodules of mature lamellar bone within a fibrous stroma, confirming the diagnosis of osseous choristoma. No recurrence was detected at the 6‐month follow‐up. This case constitutes the tenth reported instance of gingival osseous choristoma. A review of the nine previous cases is also provided to enhance diagnostic precision and clarify clinicopathologic features.

## 1. Introduction

Choristoma is a benign overgrowth of histologically normal tissue that is situated outside its expected anatomical location [1, 2]. These lesions are categorized by tissue composition and location. In the oral cavity, choristomas may contain osseous, cartilaginous, glial, lingual thyroid, gastric, respiratory, salivary, or epidermal tissues [3, 4].

Osseous choristoma is characterized by well‐formed, mature lamellar bone. It often exhibits Haversian systems and marrow spaces, and is located ectopically within soft tissue with no continuity to the native skeleton [2, 5]. Krolls et al. introduced the term “osseous choristoma” as a more specific name for these lesions. Previously, they had been called “soft tissue osteomas” or “mucosal osteomas” [5].

Intraoral osseous choristoma most commonly arises on the tongue, accounting for approximately 78% of reported cases [6]. Although lingual osseous choristoma has been well documented, gingival involvement is exceedingly rare, with only nine cases reported to date [7–15]. This report presents an additional case and reviews the existing literature, aimed at increasing diagnostic accuracy and help clinicians differentiate this uncommon lesion from its more common mimics.

## 2. Case Report

**Figure 1:**
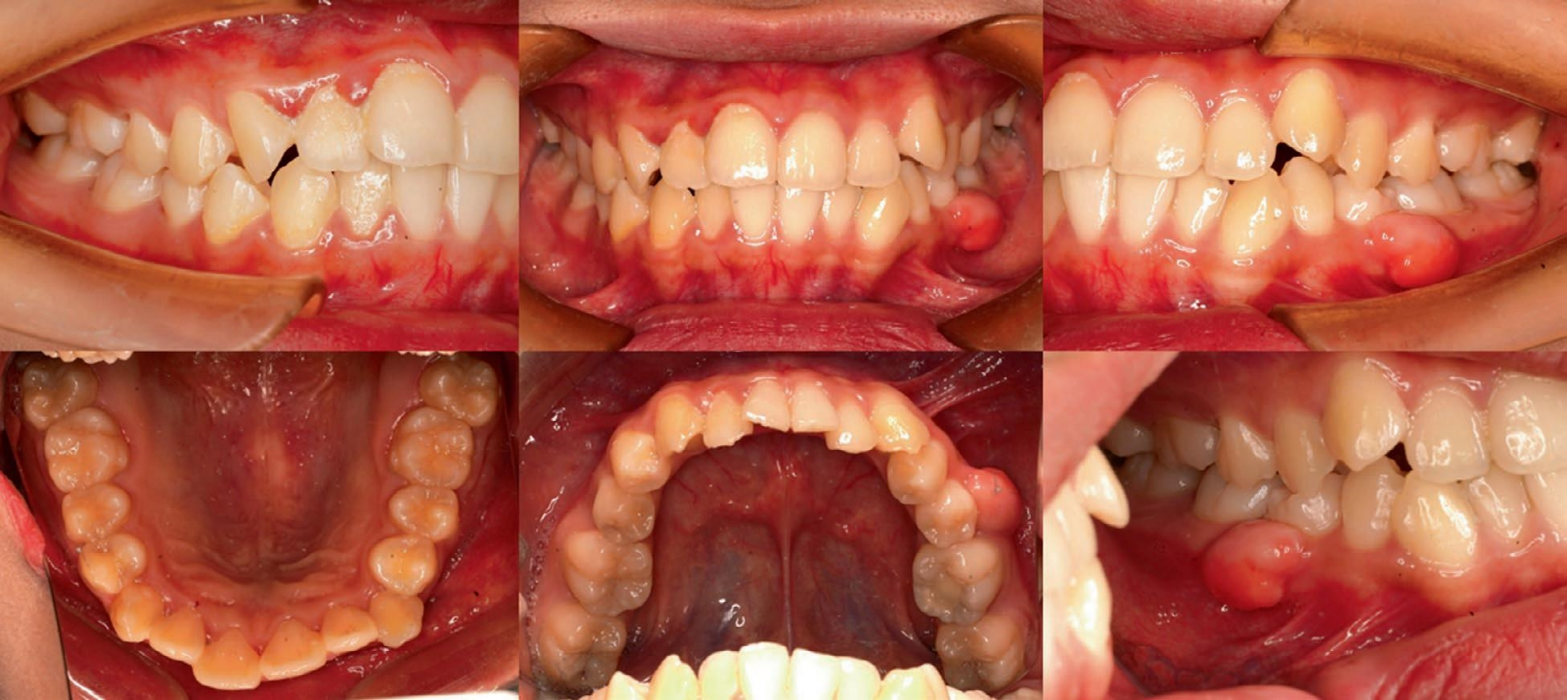
Intraoral views of the lesion.

**Figure 2:**
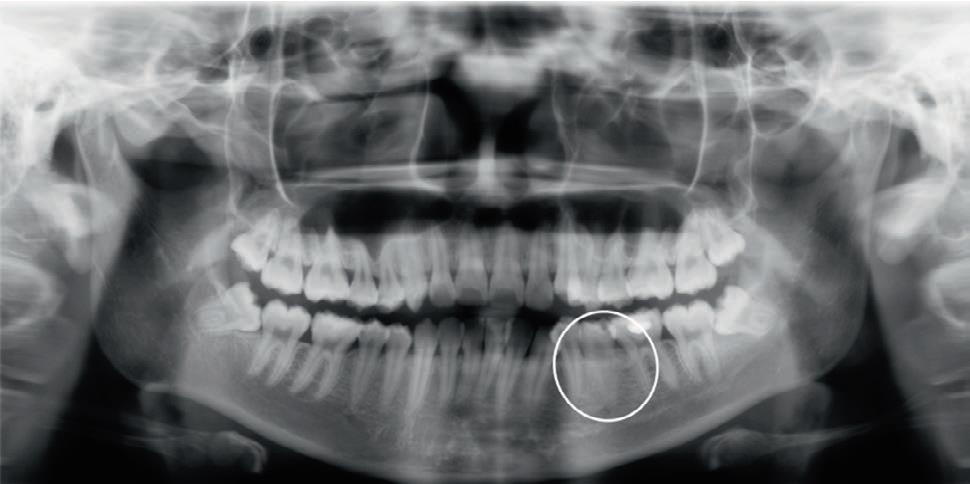
Panoramic radiography at the first visit.

A 20‐year‐old woman presented with a 6‐month history of a gradually enlarging, asymptomatic swelling in the left posterior mandible. Clinical examination revealed a well‐defined, sessile, and firm nodule measuring 1.2 × 1.0 cm on the buccal gingiva, adjacent to Tooth 35. The overlying mucosa was intact, with no erythema or ulceration (Figure 1). Tooth 35 showed buccal tipping and mesial rotation; it was vital, nontender to percussion, and nonmobile. Periodontal probing depth was 5 mm at the mid‐buccal site compared with 3 mm at all other sites. Panoramic radiography showed a faint, poorly defined radiopacity overlying the alveolar bone in the area of Tooth 35, appearing less distinct than the normal trabecular pattern (Figure 2).

**Figure 3:**
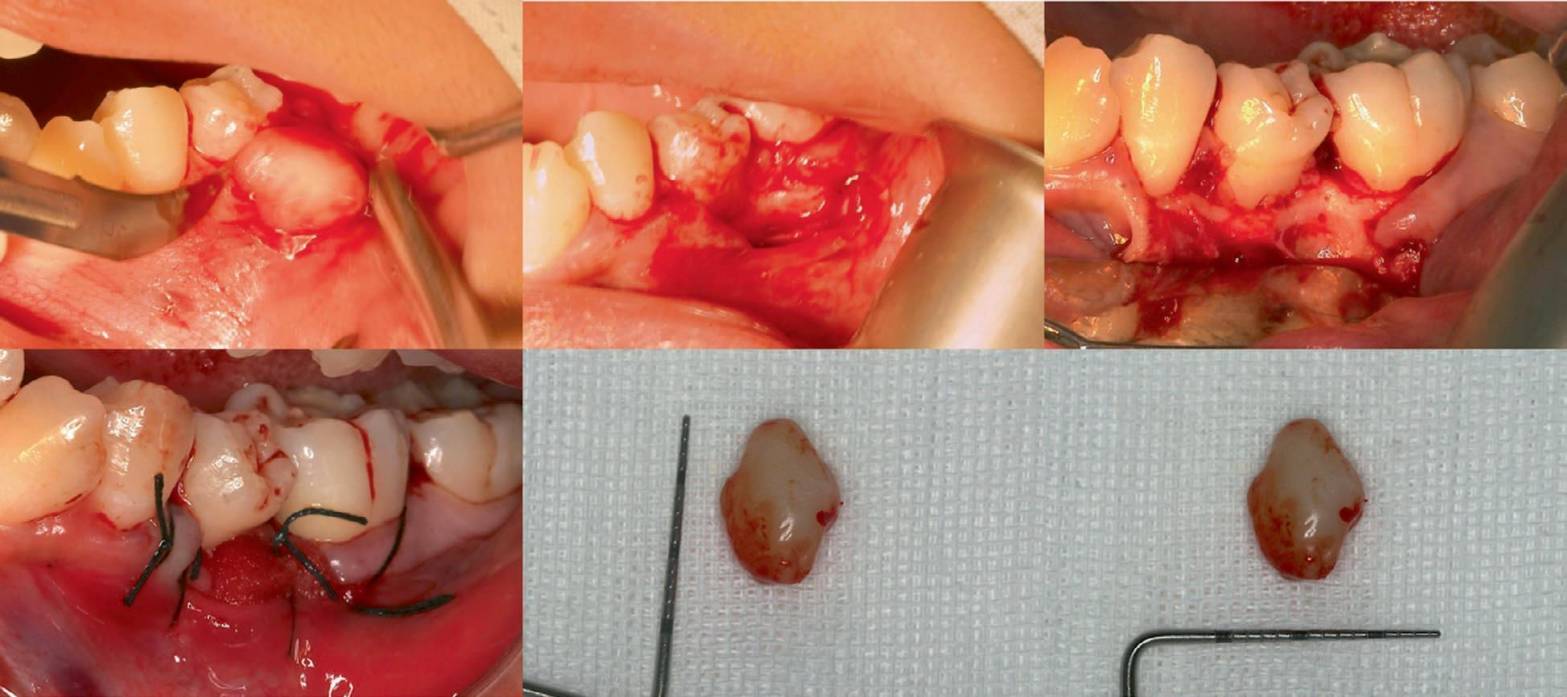
The surgical procedure for the removal of the gingival mass.

**Figure 4:**
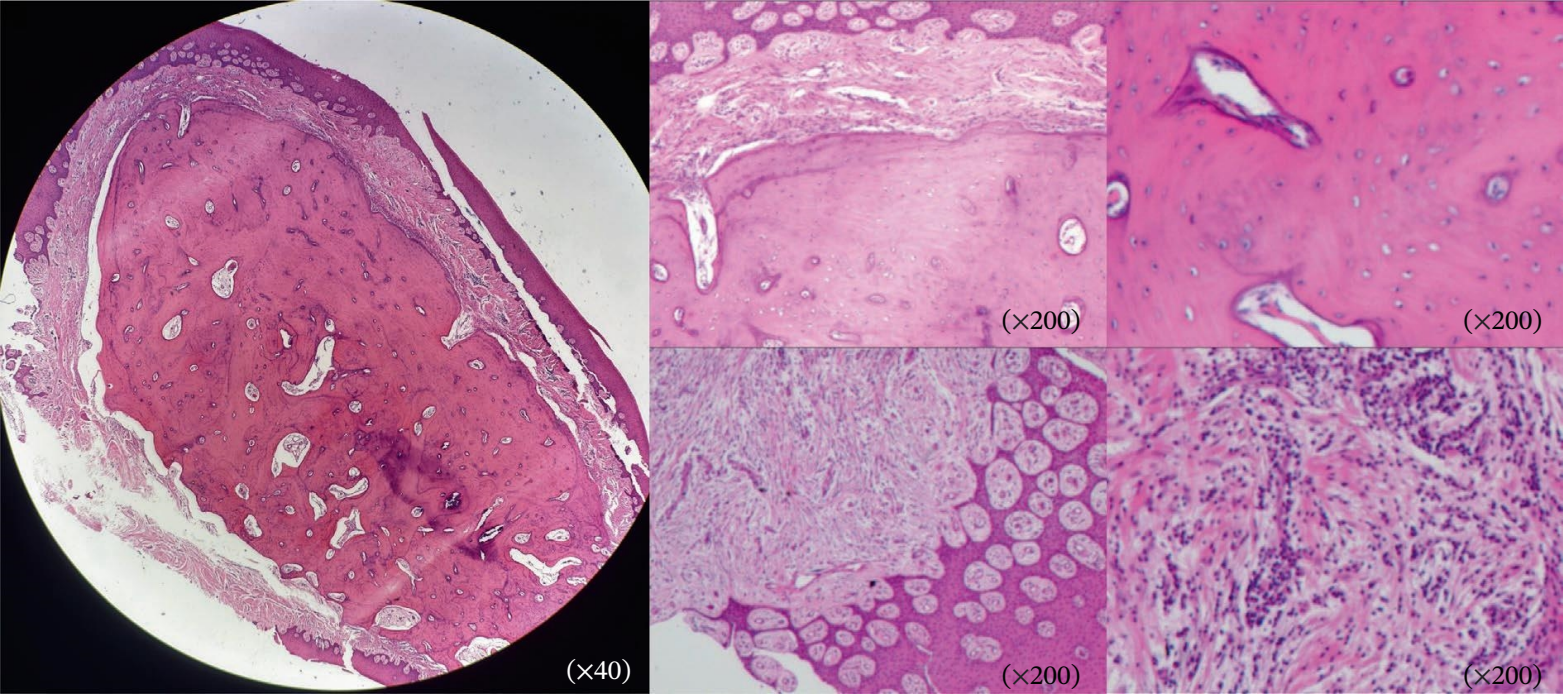
Histopathology (H&E) revealing mature lamellar bone within fibrous stroma.

The initial clinical impression was a benign fibroma. The lesion was excised at its base by sharp dissection. It was found to be firmly adherent to the gingival connective tissue, but had no continuity to or erosion of the underlying alveolar bone. The bone surface stayed intact, and no rotary instruments were needed (Figure 3). Histopathology revealed well‐demarcated nodules of mature lamellar bone with Haversian systems, embedded within a fibrocellular stroma. A mixed inflammatory infiltrate of lymphocytes, plasma cells, and neutrophils was also present. The overlying stratified squamous epithelium showed prominent rete ridges (Figure 4). These features confirmed the diagnosis of osseous choristoma. At 6 months postoperatively, no recurrence was detected.

## 3. Discussion

Osseous choristoma of the oral cavity is a rare, benign, and nonneoplastic lesion characterized by ectopic mature lamellar bone within soft tissues [1, 2]. Although the dorsal tongue is the most common site, gingival involvement is exceptionally uncommon [6]. A systematic query of PubMed/Medline and Google Scholar (1971–February 2026) employing the search strategy (“osseous choristoma” OR “bony choristoma” OR “soft tissue osteoma” OR “osteoma mucosae”) AND (“gingiva” OR “gingival” OR “alveolar mucosa” OR “periodontium”) identified only nine previously reported gingival cases [7–15], which are summarized in Table 1. This case, therefore, represents the tenth documented instance of gingival osseous choristoma. Across these 10 cases, a female predilection is noted (six females and four males), with ages ranging from 11 to 61 years. Seven lesions arose within the mandible and three within the maxilla. The average lesion diameter approximated 1.4 cm. Clinically, these lesions typically manifest as asymptomatic, slow‐growing, and firm submucosal nodules. Panoramic radiographs often yield subtle or nonspecific findings. Complete surgical excision is curative, with no recurrences reported in available follow‐up data.

**Table 1:** Gingival osseous choristoma cases presented in the literature.

Author (year)	Gender (age)	Location	Duration	Size	Base of lesion	Symptom	Radiograph	Preliminary diagnosis	Treatment	Prognosis
Sheridan (1984)	Male (61)	Lingual aspect of 31–41	15 years	1.5 cm	Pedunculated	None	NS	NS	Excision	NS
Goswamy et al. (2012)	Male (46)	Lingual aspect of 35–36	4 years	1.0 cm	Sessile	None	Well‐defined radiopaque	Peripheral ossifying fibroma	Excision	1‐year F/U, NR
Rosa et al. (2016)	Female (39)	Buccal aspect of 14	6 months	1.5 cm	Sessile	None	NS	NS	Excision	2‐year F/U, NR
Laxmidevi et al. (2019)	Male (18)	Labial aspect of 11–21	2 months	2.0 × 1.0 × 1.0 cm	Pedunculated	NS	Insignificant	Fibroma	Excision	NS
Aloua et al. (2021)	Female (39)	Right maxillary gingival	6 months	NS	Sessile	Slight pain	Normal	NS	Excision	6‐month F/U, NR
Bhakta et al. (2022)	Female (21)	Buccal aspect of 46–47	3 months	1.0 × 1.5 × 0.6 cm	Pedunculated	None	Insignificant	NS	Excision	3‐month F/U, NR
Kumbhojkar et al. (2022)	Female (61)	Labial aspect of 42–43	1 year	0.8 × 1.0 cm	Sessile	None	Insignificant	NS	Excision	NS
Pol et al. (2022)	Male (52)	Lower anterior mandibular gingiva	NS	1.5 × 1.0 × 0.6 cm	NS	None	NS	Traumatic fibroma	Excision	NS
Alhakim et al. (2025)	Female (11)	Buccal aspect of 36	1 year	1.3 × 0.8 × 0.5 cm	Pedunculated	None	Insignificant	Pyogenic granuloma	Excision	6‐month F/U, NR
Present case (2026)	Female (20)	Buccal aspect of 35–36	6 months	1.2 × 1.0 cm	Sessile	None	Insignificant	Fibroma	Excision	6‐month F/U, NR

Abbreviations: F/U, follow‐up; NR, no recurrence; NS, not stated.

This case highlights the diagnostic challenges associated with gingival osseous choristoma. Clinically, the lesion was indistinguishable from a fibroma, and panoramic radiography revealed only subtle, nonspecific findings. Localized resorption of the buccal alveolar ridge was identified intraoperatively after excision. The indistinct radiographic appearance likely results from superimposition of the calcified lesion over the resorbed ridge in a two‐dimensional image, leading to merging of structures and blurring of lesion margins. These subtle imaging findings are consistent with the literature, as small soft‐tissue calcifications may not be readily apparent on plain radiography due to their size, location, and overlap with adjacent structures [16].

Given these limitations of conventional radiography, advanced imaging modalities may be considered when a calcified gingival lesion is suspected. Cone‐beam computed tomography (CBCT) is highly recommended, as it can demonstrate a radiopaque body in the soft tissue lacking bony attachment [16]. In contrast, magnetic resonance imaging (MRI) is less sensitive for detecting calcifications. MRI can be useful for evaluating soft‐tissue relationships and for excluding other possible pathologies [17]. Importantly, despite the supportive role of these imaging modalities, none can provide a definitive diagnosis. Histopathological examination remains the gold standard for diagnosing osseous choristoma.

The differential diagnosis for a firm gingival nodule includes a broad range of calcified and noncalcified lesions [7]. The most important consideration is peripheral ossifying fibroma (POF). Clinically, both lesions present as firm gingival masses, though POF more commonly affects the anterior maxilla in young females [18]. Histopathologically, an osseous choristoma is characterized by well‐demarcated nodules of mature lamellar bone with Haversian systems, embedded in a fibrous stroma. In contrast, POF features a highly cellular fibroblastic stroma containing woven bone, cementum‐like material, or dystrophic calcification, without organized lamellar architecture [18]. This distinction—mature, organized bone versus reactive, and cellular bone formation—is diagnostically definitive.

Complete surgical excision remains the standard of care. Given the benign nature of osseous choristoma, recurrence is exceedingly rare, and no cases of malignant transformation have been reported. Isolated recurrences have been documented in the buccal mucosa (*n* = 2) and masseter muscle (*n* = 1) [19–21], but none in gingival lesions. Although no recurrence was observed in this case, the 6‐month follow‐up period is insufficient to definitively exclude late recurrence, and longer‐term surveillance may be warranted.

The pathogenesis of osseous choristoma remains uncertain. Two prevailing theories predominate: developmental displacement of pluripotent mesenchymal cells versus reactive metaplasia triggered by chronic inflammation or trauma [3, 22]. The highly organized, mature components observed in osseous choristoma are unlikely to arise solely from a reactive process, suggesting a developmental origin. In some cases, including the present one, mild peripheral inflammatory cell infiltrates may suggest a reactive component. Currently, a multifactorial model appears most plausible, in which embryologically displaced pluripotent cells undergo differentiation triggered by local microenvironmental factors, such as inflammation or mechanical stimuli. Further studies integrating developmental biology, molecular genetics, and histopathology are needed to clarify the precise mechanisms.

## 4. Conclusions

Gingival osseous choristoma is an exceedingly rare benign lesion with fewer than 10 cases documented in the English‐language literature. Its nonspecific clinical presentation as a firm, asymptomatic gingival nodule renders it clinically indistinguishable from more common entities such as fibroma or POF, posing a substantial risk of misdiagnosis. Conventional imaging is of limited diagnostic value, and histopathological confirmation of mature lamellar bone within the soft tissue remains the gold standard for definitive diagnosis. Complete surgical excision is curative. Clinicians should maintain awareness of this rare entity when evaluating gingival nodules, particularly when imaging findings are subtle or inconclusive, to ensure timely and accurate diagnosis.

## Author Contributions

Yilin Wang: writing—original draft and writing—review and editing. Chu Xu: investigation, methodology, and resources. Ting Chen: conceptualization and supervision.

## Funding

No funding was received for this manuscript.

## Disclosure

All authors assume full responsibility for the final published version.

## Consent

Written consent was obtained from the patient to publish this report and images.

## Conflicts of Interest

The authors declare no conflicts of interest.

## Data Availability

The data that support the findings of this study are available on request from the corresponding author. The data are not publicly available due to privacy or ethical restrictions.
